# Supplementary material for: Complexome profiling of the Chlamydomonas psb28 mutant reveals TEF5 as an early PSII assembly factor
Source: Plant Cell. 2025 Mar 17;37(6):koaf055. doi: 10.1093/plcell/koaf055 (PMC12164590; doi:10.1093/plcell/koaf055)
Supplement: koaf055_Supplementary_Data [file koaf055_supplementary_data.zip › psb28_interactive.zip!/plots/intensity/qualityPlots/peptides/_Cre05.g244900.t1.1.html]

### Cre05.g244900.t1.1

Trivial name:   
SeqCoverage [median]: 10.70  
Mass: 42.59 kDa  
  
 

| Sequence | MissedCleavage | Mass [Da] | wtIntensity | muIntensity | Score |
| --- | --- | --- | --- | --- | --- |
| EGADFVNR | 0 | 906.42 | 0.00 | 2705.21 | 65.25 |
| GISSLATSR | 0 | 890.48 | 1876.30 | 3495.16 | 93.16 |
| GVGDQVMTSLEK | 0 | 1262.62 | 0.00 | 2407.22 | 148.19 |
| STQLGLAVSSMLK | 0 | 1333.73 | 0.00 | 3006.81 | 106.49 |
|  |  |  | 1876.3 | 11614.4 |  |
